# Grayscale Lithography and a Brief Introduction to Other Widely Used Lithographic Methods: A State-of-the-Art Review

**DOI:** 10.3390/mi15111321

**Published:** 2024-10-30

**Authors:** Svetlana N. Khonina, Nikolay L. Kazanskiy, Muhammad A. Butt

**Affiliations:** Samara National Research University, 443086 Samara, Russia

**Keywords:** grayscale lithography, electron beam lithography, ultraviolet lithography, nanoimprint lithography, laser lithography

## Abstract

Lithography serves as a fundamental process in the realms of microfabrication and nanotechnology, facilitating the transfer of intricate patterns onto a substrate, typically in the form of a wafer or a flat surface. Grayscale lithography (GSL) is highly valued in precision manufacturing and research endeavors because of its unique capacity to create intricate and customizable patterns with varying depths and intensities. Unlike traditional binary lithography, which produces discrete on/off features, GSL offers a spectrum of exposure levels. This enables the production of complex microstructures, diffractive optical elements, 3D micro-optics, and other nanoscale designs with smooth gradients and intricate surface profiles. GSL plays a crucial role in sectors such as microelectronics, micro-optics, MEMS/NEMS manufacturing, and photonics, where precise control over feature depth, shape, and intensity is critical for achieving advanced functionality. Its versatility and capacity to generate tailored structures make GSL an indispensable tool in various cutting-edge applications. This review will delve into several lithographic techniques, with a particular emphasis on masked and maskless GSL methods. As these technologies continue to evolve, the future of 3D micro- and nanostructure manufacturing will undoubtedly assume even greater significance in various applications.

## 1. Introduction

**Figure 1:**
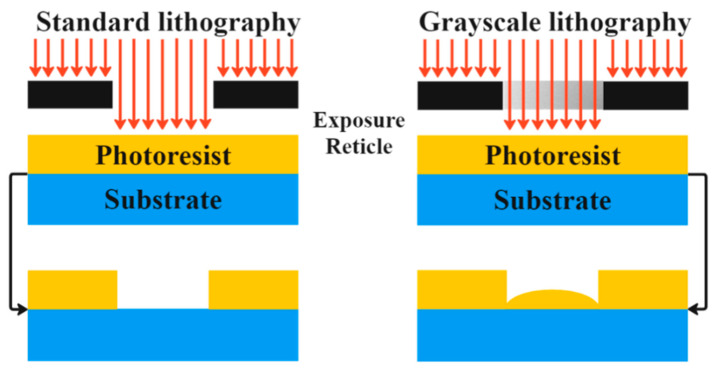
Standard lithography (**left**) versus grayscale lithography (**right**).

Standard planar lithographic techniques have been utilized since the 1960s, when the semiconductor industry began to grow, to create transistors and integrated circuit (IC) chips, which are still widely employed in modern microelectronic techniques [1,2]. The most crucial component for planar lithography is two-dimensional patterns with vertical sidewalls, as shown in Figure 1 (left). This is a technique for transferring patterns onto a substrate, typically a silicon wafer, to create the intricate circuitry that forms the basis of these devices [3]. As technology progresses, there is a relentless pursuit of electronic devices that are not only smaller but also swifter and more resourceful. Lithography serves as the catalyst for the downsizing of features on a chip, thereby facilitating the cramming of a greater number of transistors and other components into a confined space. This downsizing constitutes a pivotal element in the evolution of ever more potent and compact photonics and electronic devices [4,5,6,7].

Lithographic techniques have continuously progressed over the years, resulting in higher resolution and precision in pattern transfer [3]. Advanced lithographic methods, such as extreme ultraviolet lithography (EUVL) [8,9], X-ray lithography(XRL) [10,11], electron beam lithography(EBL) [12], and laser lithography(LL) [13], can achieve a sub-10-nanometer resolution, allowing for the fabrication of highly complex and dense patterns with accuracy and repeatability [14,15]. Achieving a high manufacturing yield is crucial in the semiconductor industry. Lithography plays a crucial role in influencing the yield of integrated circuits by specifying the key dimensions and shapes of devices. By skillfully fine-tuning and managing lithographic procedures, it is possible to reduce flaws, enhance consistency, and increase the overall production output, guaranteeing the efficient and cost-effective manufacturing of integrated circuits. Lithography has demonstrated its ability to adapt to the requirements of Moore’s Law, which dictates that the number of transistors on a chip roughly doubles every two years, making it a scalable technology [16]. As lithography evolves and improves, it enables the fabrication of smaller and more advanced devices, supporting the continued progress of the semiconductor industry [17].

Microstructures with a 3D morphology are essential to extending their potential practical applications, such as microfluidic systems, artificial eyes, and electronic skin, because of advancements in microelectronic technology, particularly the introduction of wearable devices and biosensors [18,19,20,21,22]. These distinct microstructures might also produce some intriguing phenomena, such as surface-enhanced Raman scattering, localized surface plasmon resonance, and frequency-selective surfaces [23]. The production of seamless, gently contoured surfaces represents the most challenging phase in crafting 3D microstructures, a task achievable through various techniques, such as EBL, nanoimprint lithography (NIL), capillary force lithography (CFL), and grayscale lithography (GSL). GSL has become the preferred method for creating 3D objects due to its compatibility with standard integrated circuit (IC) manufacturing processes, thorough industrial development, and the ability to achieve precise shapes when using appropriate mask designs. GSL, also known as gray-tone lithography, is a photolithographic technique that involves the fabrication of microstructures with varying depths or heights, as shown in Figure 1 (right) [24]. It allows for the creation of complex 3D structures by modulating the intensity of the light during the lithographic process [25]. GSL is used to create microlens arrays employed in imaging systems, for example, cameras, optical sensors, and projectors [26]. These arrays help in focusing and controlling the light passing through the system, improving optical performance [27].

Today, 3D nanostructures are frequently necessary for photonics and optoelectronics. Such components cannot be produced using conventional photolithography [27]. Systems from Nanoscribe, Multiphoton Optics, or Femtika, which employ femtosecond lasers and can achieve resolutions below 200 nm, enable two-photon lithography. This method can produce completely 3D structures. However, two-photon lithography is currently only capable of producing very tiny patterning volumes that are only useful for research applications, due to issues like resist shrinkage and extremely poor throughput [28]. GSL enables the fabrication of diffractive optical elements (DOEs) [29], which are used in various applications like holography, laser beam shaping, and optical signal processing [30,31,32]. DOEs manipulate the phase and amplitude of light, enabling complex light shaping and control [33,34,35,36,37]. They are also utilized to generate optical gratings used for diffraction, interference, and wavelength manipulation [38,39]. Gratings find applications in spectrometers, optical filters, telecommunications, and laser systems [40,41,42,43]. Additionally, GSL plays a crucial role in the fabrication of micro-optical components and photonic devices, including waveguides, optical couplers, beam splitters, and photonic crystals [44]. These devices find applications in optical communication, data storage, and integrated photonics. Flexible microstructures have diverse potential applications, including integrated systems, wearable technology, and biosensor electronics. Hence, it is vital to develop a practical method for producing curved and flexible microstructures. Large-scale production of distinctive 3D microstructures at a reasonable cost is still difficult despite major advancements in 2D stretchable inorganic frameworks.

In this review paper, we provide a comprehensive analysis of the latest advancements in GSL alongside a detailed examination of other widely employed lithographic techniques. GSL, with its ability to create 3D microstructures in a single exposure, plays a pivotal role in applications such as micro-optics, MEMSs, and microfluidic devices, offering enhanced design flexibility and precision. Our discussion covers both the technical innovations driving GSL forward and comparative insights into the efficiency, resolution, and versatility of various lithographic methods, highlighting their applications across different domains. Through this in-depth review, we aim to offer a clear understanding of the evolving landscape of lithographic technologies and their impact on modern fabrication processes.

## 2. Advances in GSL

**Figure 2:**
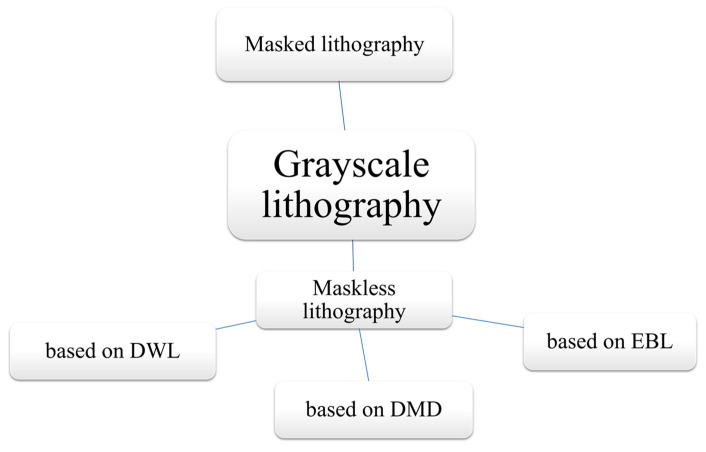
Types of GSL considered in this review.

GSL stands out as a superior lithographic technique when compared to traditional binary lithographic methods. The key advantage lies in its ability to create intricate three-dimensional structures with varying depths and heights, a feat that binary lithography cannot achieve. This versatility enables the fabrication of complex micro-optical elements, microfluidic devices, and other functional structures with exceptional precision and efficiency [27,45]. GSL also offers the advantage of dynamic control over the intensity of exposure, allowing for real-time adjustments during the patterning process, resulting in higher flexibility and adaptability. Furthermore, it excels in producing gradient-like features, which are invaluable in applications like micro-optics, microfluidics, and advanced microelectronics [18,46,47]. Researchers have developed new lithographic processes, materials, and equipment to achieve smaller feature sizes and higher precision [48]. This enables the creation of more intricate and detailed structures [49]. GSL relies on precise control of the exposure dose or development time to create different heights or depths. Recent advancements aim to improve the accuracy and reproducibility of gray-level control. This involves optimizing the exposure system, developing better calibration methods, and refining the resist materials used. In this review, we mainly focused on the recent developments in masked and maskless GSL techniques, as shown in Figure 2. The maskless GSL process was further sub-divided into three different kinds.

### 2.1. Masked GSL

In the context of GSL, the utilization of photomasks is pivotal for imprinting a design onto a substrate in the lithographic procedure. Unlike the conventional lithographic approach that relies on binary masks (black and white) to determine exposure regions, GSL mandates the use of photomasks featuring a spectrum of gray shades to yield a diverse set of exposure intensities. These intricate patterns, tailored for substrate transfer, are crafted using either computer-aided design (CAD) software or specialized lithography software. The design can encompass a variety of gray shades to attain the preferred exposure levels. Once the pattern design is ready, it must be converted into a grayscale mask file. This can be achieved using appropriate software that can assign different gray levels to different regions of the pattern. The grayscale mask file is then used to fabricate the photomask [50]. Photomasks are typically made using materials such as glass or quartz with a thin layer of chromium or another opaque material deposited on top [51]. The grayscale information is transferred onto the mask using techniques like direct writing laser (DWL) or electron beam lithography (EBL). Once the photomask is meticulously crafted, it must undergo a thorough inspection process to ensure careful reproduction of the grayscale pattern. This critical step can be accomplished through the utilization of specialized inspection tools or a close examination of the mask under a microscope. With the utmost precision, the photomask is then seamlessly integrated into the lithographic workflow, finding its place in the optical path positioned strategically between the illuminating light source and the target substrate. The fidelity of the grayscale pattern, as well as its exposure dose and resolution, are precisely regulated by the conditions of the exposure process, in conjunction with the grayscale levels meticulously defined on the photomask. It is essential to acknowledge that the intricate world of grayscale lithography is marked by its adaptability to various lithographic methods and equipment. Different technologies, such as DWL or EBL, may impose unique prerequisites and distinct workflows, reflecting the evolving landscape of lithographic innovation.

**Figure 3:**
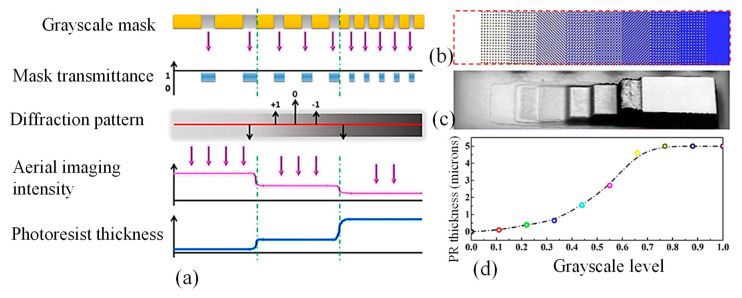
(**a**) The working principle of the GSL process [18], (**b**) ten-gray-level design for calibration [18], (**c**) optical image of the calibration sample after developing using the ten-gray-level mask [18], (**d**) their residual PR thickness for the normalized gray levels [18].

GSL may be used to create curved photoresist (PR) templates for 3D microstructures, where the PR serves as a sacrifice layer to create wave-like arching structures [18]. Figure 3a depicts the working mechanism of GSL. The optical projection may be thought of from the perspective of physical optics as a spatial filter. The item from the mask can first have its spatial frequency spectrum determined using the Fourier transform, after which it can be transmitted via an optical demagnification system with a 1:5 ratio to the diffraction surface. Due to the small numerical aperture, certain spectral orders of the object information are lost during the spatial frequency spectrum transmission process. The diffraction image is then converted to the PR layer using Fourier inversion.

The stepper’s resolution plays a crucial role in determining the maximum linewidth for GSL mask fabrication. If the pixel size on the mask exceeds the minimum resolution, it allows all spectral commands to smoothly traverse the system and reach the wafer plane. This, in turn, enables the accurate projection of fine object details onto the PR layer. However, as the size of the object increases, the loss of spectral data also increases. Conversely, when the object’s size is smaller than the minimal resolution, only zero-order spectral information remains. This results in nearly uniform light intensity on the PR layer. Consequently, this uniform light distribution can lead to the formation of curvilinear microstructures due to the gradient in light intensity. A ten-level grayscale mask with transmittance values ranging from 100% to 0% and a gradient value of 11% is shown in Figure 3b. High and low exposure dosages for UV radiation result in overexposure and underexposure, respectively, which results in the complete elimination of PR or PR residues [18]. Figure 3c demonstrates that an eight-order gray level, rather than a ten-order, is produced, at most. This is a result of the residual PR thickness and the light intensity for gray levels having a nonlinear response profile. The normalized gray levels in Figure 3d are seen to have a significant height variation between the 0.33 and 0.66 levels, demonstrating the necessity for more gray levels to achieve a smooth curving surface [18].

**Figure 4:**
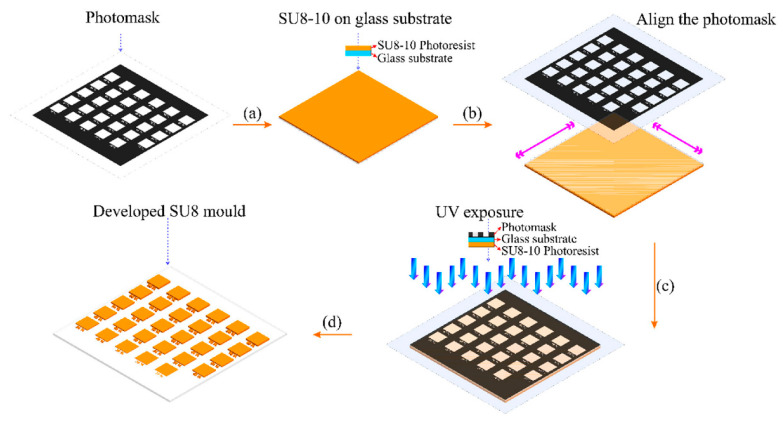
GSL process for SU-8 mold development [52].

Applications for microfluidic devices have made use of 3D microstructures. Microfluidic systems that use 3D microstructures for various functions often include multilevel structures in their design. Thus, as it has an impact on a microfluidic device’s overall performance, the efficiency of the micromixing process is essential. The present fabrication of microstructures involves slower point-to-point and layer-by-layer pattern exposure, which requires expensive and complex equipment. In [52], GSL technology is put forth that can apply a single mask and simultaneously regulate the lateral and vertical dimensions of microstructures. Depending on the length of the UV exposure, negative PR SU8 is utilized to create molds with structural heights between 163.8 µm and 1108.7 µm and grayscale concentrations between 60% and 98%, as shown in Figure 4. This method has been used in the construction of multilayer passive micromixers to analyze mixing performance. Based on an optical absorbance study, it has been shown that among different types of micromixers, the 3D serpentine shape provides the highest mixing performance.

Masked GSL offers significant advantages, particularly in its ability to create 3D microstructures in a single exposure step, eliminating the need for complex multi-step processes. This efficiency not only reduces production time but also enhances the precision of feature heights and shapes, making it ideal for applications in optics, MEMSs, and microfluidics. One of the key strengths of masked GSL is its high resolution, capable of achieving sub-micron lateral resolutions and precise control over vertical profiles. This fine control over depth and structure height is crucial for creating intricate patterns and highly functional devices at the micro- and nanoscales.

### 2.2. Maskless GSL

Maskless GSL is a technique used in microfabrication and nanofabrication processes to create patterns with varying levels of grayscale intensity, without the need for physical masks. Maskless GSL allows for greater design flexibility. Instead of using a fixed photomask with predefined patterns, maskless lithography systems can directly generate grayscale images based on digital data [31]. This flexibility enables the rapid prototyping and fabrication of complex and customizable structures without the need for time-consuming and costly mask fabrication [53,54]. In this section, three vital maskless GSL methods are discussed.

#### 2.2.1. Grayscale Electron Beam Lithography (GS-EBL)

When using GSL, it is necessary to create extremely intricate masks to locally alter the light intensity. Making such masks and ensuring that the resultant structures match accuracy standards are challenging and expensive. This GSL technique is extremely difficult to regulate and has a very small range of possible geometries, even with the right mask [55]. An alternative to mask-based lithography is grayscale electron beam lithography (GS-EBL) [56]. This technique has significantly advanced in recent years and has proven beneficial for a variety of applications, including micro-optics [57].

GS-EBL is a technique used in nanofabrication and semiconductor manufacturing to create patterns with varying levels of exposure or dose using an electron beam [58,59]. It allows for the fabrication of complex structures and high-resolution patterns with smooth gradients or continuous variations in feature sizes. The main benefit of EBL is that it has a sub-10 nm resolution for writing customized patterns. Due to its high resolution and slow throughput, this type of direct writing is only appropriate for the creation of photomasks, small quantities of semiconductor devices, and research and development [60]. Traditional EBL systems use a binary exposure method, where each pixel of the pattern is either exposed or unexposed to the electron beam, resulting in a binary pattern of exposed and unexposed areas [61]. However, GS-EBL extends this capability by allowing the electron beam to expose a pixel with a variable dose or intensity, producing intermediate levels of exposure [56].

Pixelated filter arrays composed of Fabry–Perot (FP) cavities are extensively integrated with photodetectors to realize on-chip spectral measurements that adhere to the “what you see is what you get” (WYSIWYG) principle. Nonetheless, conventional FP-filter-based spectral sensors encounter a noteworthy trade-off between their spectral resolution and operational bandwidth due to inherent design constraints associated with traditional metal or dielectric multilayer microcavities. It has been suggested that multilayer metal–dielectric-mirror Fabry–Perot (FP) microcavities used in integrated color filter arrays (CFAs) might offer hyperspectral resolution over a wider visible bandwidth (~300 nm) [62]. The FP-cavity mirror’s broadband reflectance was significantly increased by adding two more dielectric layers to the metallic film, together with as-flat-as-possible reflection-phase dispersion. This led to a balanced spectral resolution (10 nm) and a 450–750 nm spectral bandwidth. In the experiment, GS-EBL is employed as part of a one-step quick production process. A 16-channel (4 × 4) CFA was created, and it successfully displayed excellent recognition abilities and on-chip spectrum imaging with a CMOS sensor. These findings offer an appealing strategy for creating high-performance spectrum sensors and may find use in the industry by increasing the use of low-cost production processes [62].

**Figure 5:**
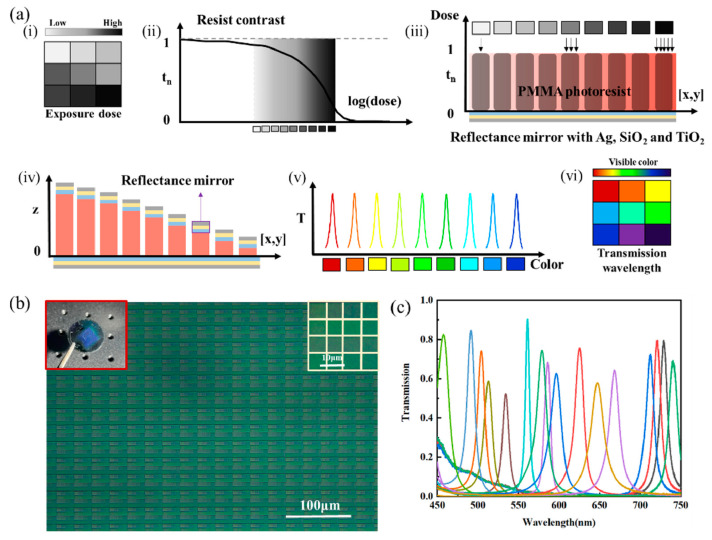
(**a**) A schematic representation of the GS-EBL process (i–iv) [62], (**b**) an optical micrograph of 16-channel CFAs operating in the transmission mode [62], and (**c**) a scale bar of 100 m. (**c**) Each wavelength band for the 16-channel system’s transmission spectrum [62].

The contrast curve was used to identify each dose-modulated spectral channel, as illustrated in Figure 5a, and the resulting transmission mode for each channel was spectrally red-shifted from 450 nm to 750 nm. The electron beam has a 100 kV output, a 300 µm aperture, and an optimal exposure dosage range of 50–148 µC/cm^2^. Following e-beam exposure, the PMMA PR was processed in a deionized water and IPA solution to produce spacer layers of varying thicknesses. PMMA color filter thicknesses range from 30 nm to 220 nm. The PMMA dielectric spacer layers with various thicknesses were then covered with symmetrical, high-reflectance mirrors. With a picture of the composite material CFAs in the upper-left, Figure 5b displays the halogen-lamp microscope pictures of the 16 channels with various thickness spacer layers. The scale bar was 100 µm, and the 16 channels (6.9 µm per pixel) were organized in a 4 × 4 matrix. Multiple spectral channels showed distinct color shifts. An Agilent CARY-5000 universal measuring spectrophotometer was then used to test the optical performance of 16 channels of filter elements with wavelengths ranging from 450 nm to 750 nm. The narrow bandwidths (10–15 nm) and normalized transmission spectra are shown in Figure 5c, where the absolute optical efficiency was close to 48% [62].

**Figure 6:**
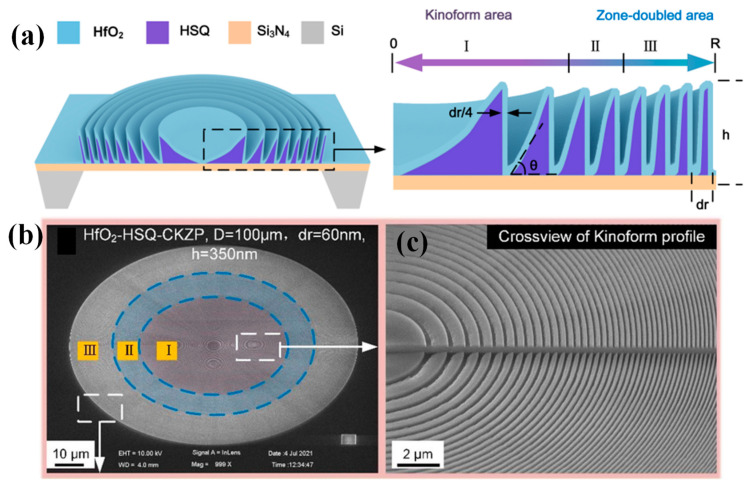
(**a**) Zone profile diagrams for HfO_2_-HSQ-CKZP. Zone-doubled HfO_2_-HSQ-CKZP images captured by SEM [63], (**b**) overviews of the HfO_2_-CKZP lens, which has a 100 µm diameter, Dr = 60 nm, h = 350 nm, and a 15 nm effective outmost zone width [63], (**c**) SEM micrographs showing the cross-sectional view of the HfO_2_-HSQ-CKZP at a 45° inclination [63].

The X-ray microscope, a crucial instrument for nanoprobing, is a key component of material nano-inspections. The efficiency of the currently available high-resolution zone plates is approximately 5% in soft X-ray and rapidly drops to 1–2% as the resolution approaches 10 nm, despite the significant advancements of high-resolution focusing/imaging documented. It is commonly recognized that the rectangular form of the zone, the beamstop, the constrained height/width ratios, the material’s ability to absorb light, and structural flaws are probably to blame for the low efficiency. Although zone plates with the Kinoform profile are claimed to be effective, soft X-ray has made very little progress toward obtaining both a high resolution (30 nm) and high efficiency (>5%). It has been suggested that we should combine a dielectric Kinoform zone plate with a 15 nm resolution to create a composite Kinoform/Fresnel zone plate (CKZP) [63]. The binary zone plate was created using atomic layer deposition, while the 3D Kinoform zone plate was created using GS-EBL. Optical characterizations showed that focusing and imaging with a 15 nm resolution could be performed with more than 7.8% efficiency in soft X-rays. The CKZP with the radius of R is shown in Figure 6a, and the Kinoform zone plate with the outermost zone width of 15 nm is shown in purple. This plate was created using EBL on hydrogen silsesquioxane (HSQ) based on SiOx [63]. Figure 6b shows a manufactured HSQ-Kinoform zone plate with a 100 µm diameter and an outermost zone width of 30 nm at a low SEM magnification. Figure 6c also shows an SEM picture of the inner zones with a profile resembling a Kinoform [63].

GS-EBL offers significant advantages in terms of precision and flexibility for creating complex 3D micro- and nanostructures. One of its key benefits is the ability to modulate exposure doses, allowing for smooth, continuous height variations across a resist layer. This precise control enables the fabrication of intricate structures such as micro-optics, MEMSs, and advanced photonic devices. GS-EBL also provides an exceptional resolution, often achieving sub-10-nanometer accuracy, making it ideal for applications requiring fine structural detail and precise depth control at the nanoscale. Its versatility and high resolution make GEBL a powerful tool in next-generation nanofabrication.

#### 2.2.2. GSL Based on a Digital Micromirror Device

For the development of microstructures, maskless photolithography was suggested two decades ago [64,65]. The conventional physical mask has been replaced with a digital micromirror device (DMD). An array of micromirrors that can be individually controlled by a computer make up a DMD, allowing for the real-time dynamic display of a digital picture functioning as a mask [66]. The absence of the need for a costly physical mask is one of the major benefits of maskless photolithography. According to the profile of the computer-fabricated structure, the digital image can be a flexible design. The area of microfabrication development has seen a lot of attention paid to maskless lithography because of its capacity to be both inexpensive and very adaptable [67]. The following are the primary fabrication techniques used to date to create micro-optic components using maskless photolithography: The first step is serially slicing the CAD data of the structure’s surface function in the high direction, with each slice representing a binary picture. The imaging system subsequently delivers these binary pictures to the surface of the PR, where a superimposed exposure dosage proportionate to the profile function is achieved [68]. These binary images are created by the DMD under computer control in real-time [69]. This method was used by Totsu et al. to create positive PR patterns for spherical and aspherical microlens arrays, each with a 100 µm diameter [70]. The method has also been used by Zhong et al. to create continuous relief micro-optic components [71]. Even though these works may produce micro-optic components quickly and inexpensively, a drawback of these techniques is the time-consuming preparation of slicing for each design.

**Figure 7:**
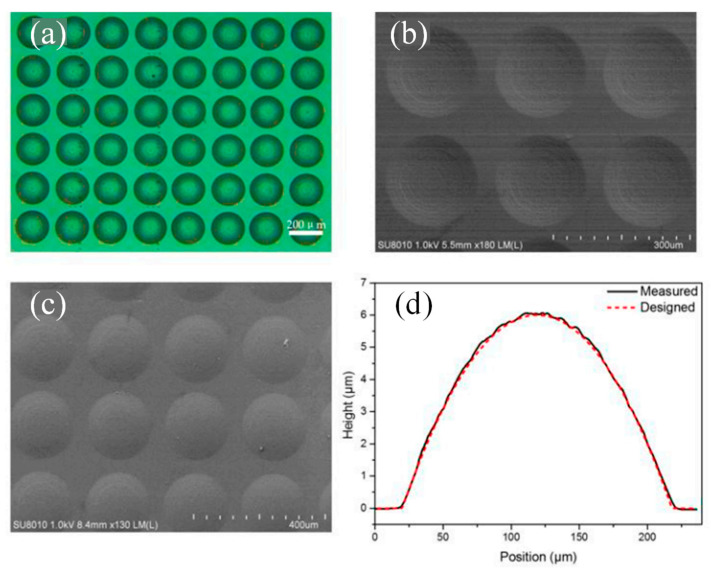
(**a**) Microscope image of a concave spherical MLA in PR [72], (**b**) SEM image of a concave spherical MLA in PR [72]; (**c**) SEM image of a convex spherical MLA in PDMS [72]; and (**d**) measured and designed cross-sections of the convex spherical MLA [72].

A method for quickly and affordably fabricating micro-optic components with arbitrary surface profiles using maskless lithography was put forward [72]. To adapt the exposure dose to the surface profile of the forthcoming structure, a DMD has been used. The dependence of the exposure depth on the exposure dose as well as the relationship between the grayscale levels of the DMD and the exposure dose on the surface of the PR deviate from a linear relationship arising from the DMD and PR, respectively, and cannot be systemically eliminated, meaning complicated fabrication techniques and significant fabrication errors will result. To precisely construct the digital grayscale mask and guarantee perfect control of the surface profile of the structure to be manufactured, a technique of balancing the two nonlinear effects is provided. Several typical array elements with spherical, aspherical, and conic surfaces have been created and tested as evidence of the validity of this methodology. This concave spherical microlens array (MLA) in the PR can be seen in the microscope and SEM pictures in Figure 7a,b, respectively [72]. The PR mold in Figure 7b was reversely transferred into the PDMS elastomer to produce the readily accessible spherical MLA. The mold was then coated with the PDMS solution, which was heated to 100 °C for 15 min. Convex spherical MLA on the surface of PDMS can be seen in the SEM image form in Figure 7c [72]. A cross-section of the convex lens was profiled using the stylus profiler, as can be seen in Figure 7d, to assess the surface profile of the PDMS MLA [72].

**Figure 8:**
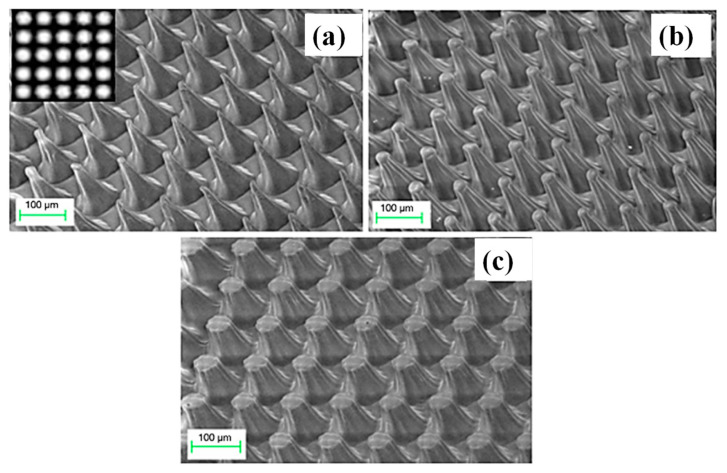
Truncated cone/needle-like patterns manufactured using different exposure times: (**a**) 400 s, (**b**) 800 s, and (**c**) 1200 s (inset shows the software mask used) [73].

Gray-tone masks (GTMs) are typically used to create GSL. The ability to mass produce micromachines with different topographies is made possible by GTMs, which allow different quantities of light to flow through. Depending on the parameter one wants to improve, GTMs are preferable to software masks or vice versa. E-beam and laser writing are slow serial approaches. They also need expensive equipment to operate. On the contrary, great throughput may be achieved when using parallel methods that expose a broad region all at once and make use of physical gray-tone masks. A new mask is needed for each pattern, which makes the manufacture of GTMs a time-consuming and expensive procedure. Consequently, using a software mask is preferred since it provides design freedom. Therefore, a software-based approach with high fabrication throughput is preferred. The use of maskless GSL has been suggested as a unique and straightforward technique for fabricating intricate 3D structures in SU-8 PR [73]. The suggested technique modulates the light intensity across a single SU-8 PR layer using a digital micromirror device (DMD^®^). When creating unique structures like cantilevers, covered channels with embedded features, and arrays of microneedles, top and backside exposure is used. With alternative methods, it is sometimes necessary to design many stacked layers or use sophisticated physical masks to fabricate equivalent structures in SU-8. The SEM images of the truncated cone/needle-like patterns taken at 400 s, 800 s, and 1200 s of exposure time are displayed in Figure 8a–c, respectively. From the photos, it can be seen that 400 s exposure intervals produce sharp, needle-like structures as tall as the coated SU-8 film’s thickness (120 µm). The needle-like cones gradually start to become truncated and then adopt the appearance of mesas when the exposure duration is prolonged from 400 s to 800 s and finally to 1200 s. Even in areas with low RGB values, an increase in exposure duration produces enough exposure energy to polymerize the SU-8 film [73].

DMD GSL typically operates with limited grayscale levels, often around 8 to 16 levels due to the inherent binary nature of DMDs. These devices consist of an array of micromirrors that can be individually tilted to either reflect light towards the substrate (ON state) or away from it (OFF state). The binary operation of DMDs restricts the number of discrete grayscale levels that can be achieved, as each micromirror can only be in one of these two states. While DMDs offer high precision, their tilt angles may not provide the fine-grained control required for a wide range of grayscale levels. Implementing more intricate analog micromirrors with an extensive grayscale range would not only complicate the technology but also significantly increase its cost and potentially reduce the resolution.

This limited resolution can restrict the ability to create highly precise and smooth gradients or complex 3D structures. This technique is better suited to simple patterns with relatively low complexity. As the complexity of the pattern increases, it becomes more challenging to accurately reproduce the desired design due to the limited grayscale resolution and potential patterning artefacts. DMD GSL relies on the interference of light to generate the desired patterns. Diffraction effects may cause unwanted features and decreased accuracy in fabricated structures, especially when dealing with smaller features or intricate optical setups. Aligning multiple grayscale exposures precisely is challenging, particularly for complex patterns or large-scale fabrication, and misalignment can lead to distorted or inaccurate structures.

#### 2.2.3. Direct Writing Laser Grayscale Lithography (DWL-GSL)

**Figure 9:**
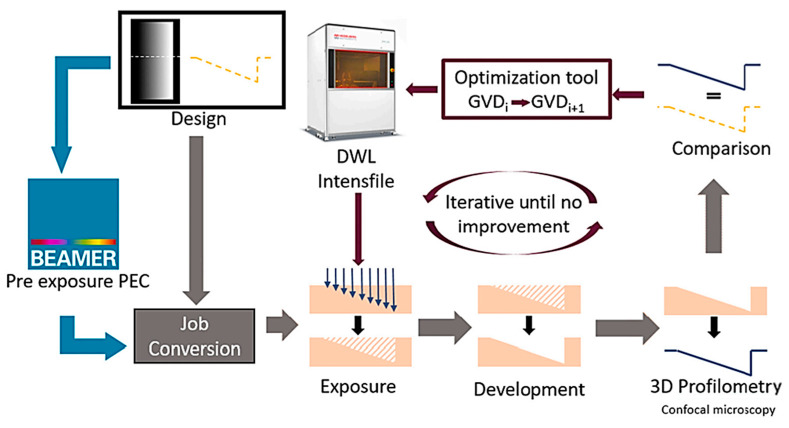
Process flows of both optimization methods used. The arrows indicate the approaches: violet (GVD iterative) and cyan (PEC model) [49].

Advanced technologies for converting design layouts into resist structures encompass laser-based pattern generators. When crafting structures with diverse topographies, whether sloping, stepped, or continuous, it becomes imperative to address the nonlinear response and proximity effects stemming from the laser beam’s interaction with the low-contrast PR. To examine the topological variances between the measurement findings and the gray-tone design, cross-sections were collected [49]. While a local correction of dose values using mathematical models is favored for more complicated geometries, an iterative adjustment of the global dose distribution is suitable for simpler shapes. One-step exposure of the corrected map necessitates a thorough evaluation of a contrast curve and an understanding of several factors. The capacity of current model-based techniques to optimize form toward the goal design within one cycle was shown by the coefficient of determination R^2^ as a unitless single figure of merit in the quantitative comparison of the two methods [49]. The process flow of both optimization processes is shown in Figure 9 [49].

Controlling the local exposure of the resist with extreme precision is essential for GSL. The approach used in Heidelberg Instruments’ DWL (direct write laser) systems (Heidelberg Instruments Mikrotechnik GmbH, Heidelberg, Germany) illustrates how this is possible [74]. These devices’ optical “hearts”—a spatial light modulator made up of an acousto-optic modulator (AOM) and an acousto-optic deflector (AOD)—allow for grayscale patterning. AOMs are piezoelectric transducers coupled to acousto-optic crystals that are powered by radiofrequency electric signals. The crystal perceives these vibrations as sound waves, causing localized changes in its optical density. Consequently, the passing light is subject to diffraction, potentially affecting the overall light intensity. This approach offers an extensive spectrum of grayscale values due to its fully analog nature. Following its passage through the AOM, the light enters the AOD. The system incorporates both a piezoelectric transducer and an acousto-optical crystal. The laser beam travels in a unidirectional path while the frequency of the acoustic oscillations is modulated. As a result, the writing field is simply a stripe, and the spatial light modulator scans the substrate “line by line” while defining the exposure depth by adjusting the laser light intensity [45]. The final result’s vertical resolution is constrained by the surface roughness. There is no relationship between the number of gray values utilized and process throughput because all of them are revealed in a single phase.

**Figure 10:**
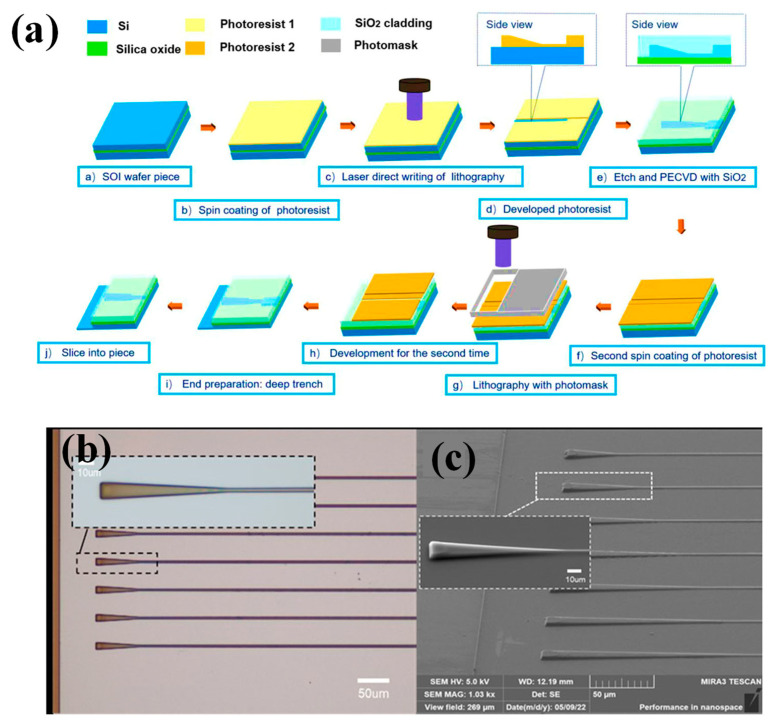
(**a**) Fabrication process [76], (**b**) microscope image of taper and semi-conical edge coupler [76], (**c**) SEM image of taper and semi-conical edge coupler [76].

The fabrication of silicon-glass nanofluidic devices with non-uniform channel depths in the 20–500 nm range and µm resolution in width has been described. Reactive ion etching (RIE) was used to transfer the PR depth profile onto silicon after the GSL DWL stage, which structures the PR in 2.5 dimensions. It was simple to incorporate into a detailed process flow chart. The technique was employed to create a network of linked, irregularly spaced slits with a shape that resembles a nanoporous medium [75]. On a 3.5 µm silicon device layer of a silicon-on-insulator, two single-mode fiber-to-chip 3D edge couplers were presented, each with taper and semi-cone structures [76]. DWL GSL and ICP-RIE were the methods used to create 3D edge couplers. The greatest coupling effectiveness of 3D couplers in the TE mode, according to experimental data, is roughly 83.41%. The schematic of the fabrication process is shown in Figure 10a, and the detailed process has been explained in [76]. The microscope and SEM images of the taper and semi-conical edge coupler are shown in Figure 10b,c, respectively [76].

With DWL-GSL, a substrate larger than 1 m^2^ may be printed for industrial use. Such a big region may need many days to be completely exposed. The DWL-GSL must be scaled up to fit industrial production processes. The speed of the light modulators might be increased, and parallel exposure could be made possible, as potential methods to increase throughput. Mask aligners or steppers are already being replaced by DMD in higher-throughput direct-write systems used for 2D applications. Similar techniques could be used in future GSL equipment. GSL with sufficiently high throughput will eliminate the necessity of replicating procedures, much like in the case of mask aligners for 2D. Currently, high-throughput manufacture of 3D microdevices still requires injection molding and NIL [27].

DWL-GSL offers significant advantages for fabricating 3D microstructures with precise depth control. This technique uses a focused laser beam to directly modulate the exposure intensity on a photosensitive material, enabling smooth, continuous variations in surface topography without the need for masks. DWL-GSL is highly versatile, allowing for the rapid prototyping of complex structures such as micro-optical components, microfluidic channels, and MEMS devices. It provides good resolution, typically reaching micrometer to sub-micrometer scales, making it ideal for applications that require accurate control over both lateral and vertical dimensions in 3D patterning.

### 2.3. Materials for GSL

Hybrid materials are currently popular due to their strong chemical resistance and resilience to temperature changes and mechanical stress. Photopolymer hybrid organic–inorganic compositions have been extensively researched in the past two decades for replicating optical microstructures [77] and creating relief patterns with laser and photolithographic technologies [78]. Structures made from these materials are notably more mechanically robust compared to standard positive photoresists like AZ15XX and S18XX used in multilevel laser writing and photolithography. The Ormocer hybrid polymer series, developed by the Fraunhofer Institute for Silicate Research ISC, is a well-known and widely used group of hybrid photopolymer materials, including Ormocomp, which serves as a negative photoresist for crafting various optical components [79]. In the process of replicating a structured original, photopolymerization is performed using a broad and evenly intense ultraviolet light beam. Additionally, it is possible to create binary microstructures (with two relief levels) on Ormocer materials through direct laser writing or photolithographic exposure using a photomask [80].

To create a multilayer profile in the layers of a Hybrimer-TATS hybrid photopolymer material based on thiol-siloxane and acrylate oligomers, an optical technique investigation has been conducted [81]. Multilevel structures measuring 3.5 and 6 µm in height are created using GSL and direct writing laser (DWL), respectively. The material’s characteristic curves and photosensitivity are established. The techniques for preparing and processing the film are optimized, and it is found that the inclusion of pre- and post-exposure steps has a substantial impact on the photosensitive characteristics of Hybrimer-TATS [81]. It has been shown that several essential elements make it feasible to create 3D photonic integrated circuits (PICs) utilizing polymers [82]. This enables us to fabricate intricate 3D integrated optic structures using shadow reactive ion etching, shadow photolithography, and GSL. Vertical power splitters have predictable output splitting ratios across numerous core levels with excess losses of 0.5 dB, whereas vertical waveguide bends have excess losses of <0.3 dB. Power extinction ratios of 15 dB are seen in vertical polarization splitters between the output core layers. Additionally, a 1 × 4 vertical–horizontal power splitter has been demonstrated. Additionally, these methods are employed to conveniently address the mode mismatch issue while integrating several polymer materials into the same optical circuit [82].

Nanotextures are becoming increasingly significant in nanotechnology, with recent studies showing that their functionality can be greatly enhanced by spatially modulating the height of nanoscale pixels. However, achieving this “grayscale” modulation—where the pixel height is controlled with nanometer-level precision as a function of position—presents substantial challenges. Li et al. addressed those challenges by demonstrating a highly precise method for grayscale printing of polymeric nanopixels, offering both high vertical and lateral resolutions [83]. The key to this advancement lied in controlling the capillary rise of specific photopolymers through light exposure, allowing for a sub-10-nanometer accuracy in height modulation. By spatially patterning the control light, the technique enabled 2D grayscale nanopixel printing. The effectiveness of this approach was validated through reconfigurable, maskless printing of grayscale nanopixel arrays in both dielectric and metallic–dielectric forms. Additionally, the study uncovered the highly nonlinear and unstable characteristics of the polymeric nanocapillary effect, broadening its potential applications and deepening the understanding of this phenomenon [83].

Jeon et al. proposed a novel approach to control micropillar height using the same mold by adjusting the cavity size and the viscosity of photocurable polyimide resin [84]. Smaller microcavities or higher resin viscosity resulted in shorter micropillars. Additionally, micropillar arrays were demonstrated, which can be arbitrarily patterned in height through the application of masking techniques. This cost-effective and flexible method holds promise for a range of advanced applications, including metasurfaces for electromagnetic signal manipulation and biomedical uses such as cell culture and stem cell differentiation [84].

## 3. Challenges Associated with GSL

Achieving precise and high-resolution grayscale masks can pose a considerable challenge. Conventional lithographic methods typically rely on binary masks, which consist of distinct transparent and opaque regions. However, GSL demands masks with varying levels of transparency, making the fabrication of such masks technically intricate and time intensive. Furthermore, GSL techniques may inherently struggle to attain an exceptionally high resolution. This achievable resolution is influenced by multiple factors, including the wavelength of light employed, the numerical aperture of the optics, and the characteristics of the photoresist (PR) material. Overcoming these limitations to achieve submicron or even nanoscale features can prove to be a formidable task. In the quest for optimal GSL performance tailored to specific applications, the complexity further escalates. Critical factors like exposure dose, development time, and post-processing steps must be meticulously controlled to attain the desired feature dimensions and profiles. Fine-tuning these parameters for various materials and device designs becomes a labor-intensive and iterative process. GSL opens the door to crafting intricate 3D structures with varying depths, but mastering precise depth control presents its own set of challenges. Factors such as the thickness and uniformity of the PR layer, exposure dose, and development characteristics wield a profound influence over the final depth profile. Striving for a high degree of fidelity in replicating the intended grayscale pattern can be a formidable endeavor. Furthermore, GSL often demands specialized equipment and infrastructure, necessitating a substantial financial commitment for establishment and upkeep. The requisite GSL equipment, encompassing high-resolution photomask writers and exposure systems, may demand significant capital investments. Beyond the financial considerations, GSL may entail additional procedural steps and extended processing times when juxtaposed with conventional lithographic techniques. This expanded complexity can engender a heightened overall cost of implementation. GS-EBL achieves a high resolution, but it is still limited by the beam size of the electron beam. The achievable resolution depends on factors such as the beam current, accelerating voltage, and beam diameter. However, even with advanced techniques, the resolution is generally limited to the nanometer scale, making it less suitable for applications requiring sub-nanometer resolutions or extremely fine features. GS-EBL can be a time-consuming process due to the need for precise scanning of the electron beam over the substrate. The process typically involves scanning the electron beam pixel by pixel to expose the desired pattern, resulting in relatively slow writing speeds. This can limit the throughput and efficiency of GS-EBL, making it less suitable for high-volume production or large-area patterning. Moreover, the proximity effect is a significant limitation in GS-EBL. This refers to the phenomenon where electron scattering and secondary electron generation from nearby exposed regions affect the exposure dose and result in unintended blurring or distortion of the desired pattern. The proximity effect becomes more pronounced as feature sizes decrease, making it challenging to achieve high fidelity and accuracy in the fabricated structures. Electron beams can cause charging effects on insulating or poorly conducting substrates. The accumulated charge can influence the trajectory of subsequent electrons, leading to distortions or deviations from the desired pattern. Charging effects can be mitigated through various techniques such as conductive coatings or charge compensation, but they can still impact the quality and uniformity of the fabricated structures.

GSL offers an intriguing avenue for various applications, but it is not without its alternatives and challenges. Instead of conventional electron beam lithography (EBL), GSL has been compared to alternative methods like focused ion beam (FIB) milling and focused electron beam-induced deposition (FEBID). While GSL, FIB, and FEBID all bypass the need for wet development and permit the direct patterning of 3D nanostructures, they share common drawbacks. Both FIB and FEBID exhibit sluggish processing speeds, and they tend to introduce substrate contamination. Achieving single-nanometer 3D precision remains an elusive goal with these techniques, limiting their widespread adoption. Furthermore, these procedures are costly and technically demanding, presenting additional hurdles.

Although GSL based on DMD technology boasts a higher resolution compared to traditional lithography (operational with arc lamp or mercury lamp) methods, it grapples with inherent limitations. The attainable feature size is contingent on factors such as the DMD’s pixel size and pitch, as well as the optical components integrated into the system. This makes GSL unsuitable for applications requiring ultrafine features in the sub-micrometer or nanometer range. Additionally, GSL via DMD is susceptible to diffraction effects that can compromise the accuracy and quality of fabricated patterns. Diffraction phenomena can manifest as blurring, interference, and sidelobe artifacts, particularly when handling intricate structures or minuscule features. To counter these diffraction-related issues, advanced optical techniques and optimization strategies are imperative.

Conversely, the achievable resolution in direct writing laser grayscale lithography (DWL GSL) is contingent upon several factors, including the size of the laser beam spot, the presence of diffraction effects, and the optical configuration employed. When dealing with diminutive features or intricate designs, attaining high-resolution features can prove to be a formidable task. These resolution constraints may exert an influence on the level of intricacy and precision attainable in grayscale patterns. Accurate grayscale pattern generation necessitates meticulous laser beam scanning and precise positioning. Any deviations or misalignments during the scanning process can lead to distorted or inaccurate features. Ensuring elevated scanning precision and maintaining exact beam positioning over expansive areas can present technical challenges, often necessitating the deployment of advanced scanning systems.

DWL GSL relies on the interaction between the laser beam and the substrate material to craft grayscale patterns [85]. Nonetheless, not all materials harmonize seamlessly with laser lithography. Certain materials may exhibit distinct behaviors in terms of laser energy absorption or reflection, resulting in variations in the grayscale response [86]. Consequently, prudent material selection is imperative to guarantee a robust grayscale performance and dependable fabrication outcomes [87]. Furthermore, DWL GSL frequently mandates specialized equipment, encompassing high-powered lasers, sophisticated scanning systems, and meticulous control mechanisms. The cost associated with procuring and operating such equipment, along with the intricacies involved in their maintenance, can pose substantial challenges, particularly in the context of modest-scale research initiatives or prototyping environments. Despite these formidable challenges, GSL retains its value across numerous applications. It particularly shines in the realm of micro-optical elements, diffractive optics, microfluidic devices, and other intricate microstructures. The ongoing focus of research and development endeavors revolves around surmounting these challenges and enhancing the capabilities and accessibility of GSL.

## 4. Other Lithographic Methods

**Figure 11:**
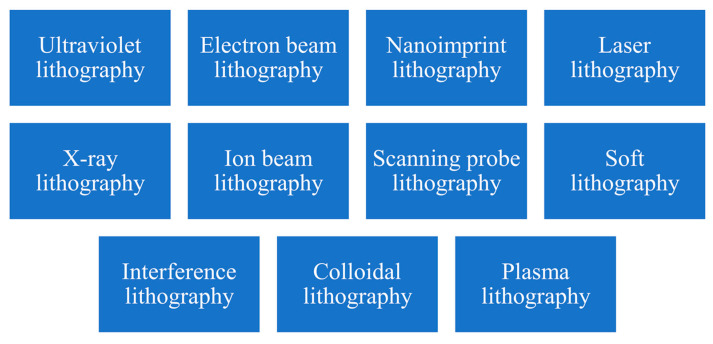
Other noteworthy lithographic methods.

Over the years, various lithographic methods have been developed to achieve increasingly smaller feature sizes and higher levels of complexity [12,38]. In this section, some commonly used lithographic methods, as shown in Figure 11, are briefly discussed.

### 4.1. Ultraviolet Lithography (UVL)

**Figure 12:**
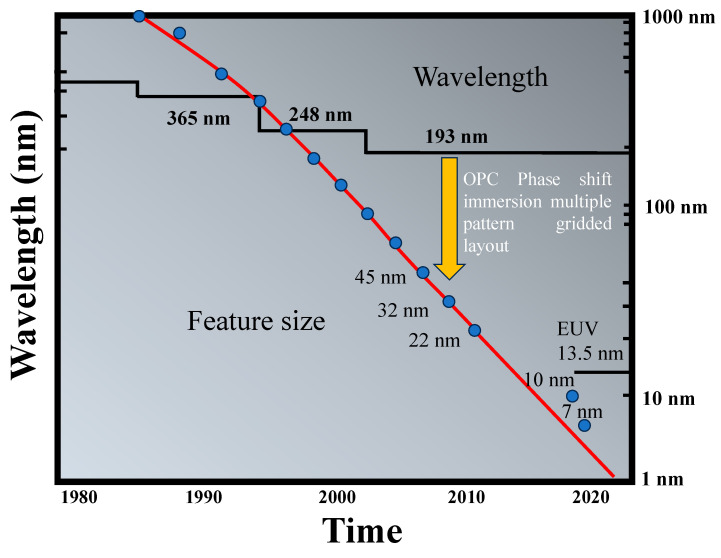
Historical progression of IC feature size and photolithographic technologies. Inspired by [91].

UVL has been a fundamental technology in semiconductor manufacturing for several decades. It enables the production of smaller and more densely packed features on chips, allowing for increased functionality and performance. UVL benefits from mature infrastructure, large-scale manufacturing capabilities, and cost-effective equipment, making it suitable for high-volume production. As technology advances, the industry has been pushing towards even shorter-wavelength light sources, such as extreme ultraviolet (EUV) light, to achieve finer feature sizes and higher levels of integration [88,89,90]. Figure 12 provides a comprehensive overview of the historical evolution of integrated circuit (IC) feature sizes and the corresponding wavelengths of photolithography light sources essential to achieve these diminishing feature dimensions. For a substantial part of its history, the realm of photolithography was predominantly preoccupied with the relentless pursuit of reducing the wavelength of the light source to enable the fabrication of ever-smaller features. The chronological progression unfolded as follows:

- Arc Lamp Systems (1970s and Early 1980s): These pioneering systems, with a wavelength (λ) of 436 nanometers (nm), were instrumental in enabling feature sizes as small as about 450 nm.
- Mercury Lamp I-Line Systems (Mid-1980s): Emerging in the mid-1980s, these systems operating at a wavelength of 365 nm allowed for further feature size reduction, reaching approximately 380 nm.
- KrF Excimer Laser-Based Systems (1990s): Stepping into the 1990s, lithographic technology took a significant leap with KrF excimer laser-based systems, operating at a wavelength of 248 nm. These systems advanced the industry, achieving feature sizes of about 250 nm.
- ArF Excimer Laser-Based Systems: The modern era ushered in ArF excimer laser-based systems, characterized by a remarkably short wavelength of 193 nm. These systems demonstrated their prowess by enabling feature sizes to shrink down to a striking 65 nm.

Modern deep ultraviolet (DUV) photolithography systems use ArF excimer lasers with a wavelength of 193 nm. These systems, when using air as the medium between the optical system and the substrate (NA = 1), would typically be limited to feature sizes of 65 nm or larger. To achieve smaller feature sizes for technological nodes like 45 nm, 32 nm, and 22 nm, advanced techniques were necessary. Manufacturers employed a combination of optical proximity correction (OPC), phase shift, immersion lithography, and multiple patterning to push the boundaries of 193 nm lithography and achieve smaller feature sizes than conventionally expected. Dry 193 nm photolithography in conjunction with double patterning successfully achieved 45 nm patterning technology. This technology was further improved with the incorporation of immersion lithography to achieve 32 nm (and smaller) patterning technology.

OPC techniques were instrumental in compensating for image distortions that arise when printing features smaller than the wavelength of the exposing light. These distortions typically lead to issues such as line end shortening, corner rounding, or changes to linewidth. OPC corrections were implemented at the mask level and involved modifications to the mask image, including the addition of serifs at design corners and adjustments to linewidths.

This historical trajectory exemplifies the relentless drive within the semiconductor industry to push the limits of photolithography and attain ever-smaller feature sizes, a pursuit enabled through the continuous reduction in the light source’s wavelength. In UVL, a photosensitive material, called a PR, is applied to a substrate, typically a silicon wafer. PR is sensitive to ultraviolet light, specifically short-wavelength ultraviolet (UV) light. A mask, which contains the desired pattern for the circuit, is placed over the PR-coated wafer. Ultraviolet light is then shone through the mask, exposing the PR in specific areas according to the pattern. The exposed regions of the PR undergo a chemical change, becoming either more soluble or less soluble in a developer solution, depending on the type of PR used. After the exposure, the wafer is immersed in a developer solution, which selectively removes either the exposed or unexposed portions of the PR, depending on the type of resist used. This step transfers the pattern from the mask onto the wafer. Once the PR has been developed, subsequent processes such as etching, deposition, and implantation are performed to create the desired circuit features on the wafer. The UV lithographic step is repeated multiple times, using different masks, to build up the complex layers and structures required for the integrated circuit [92].

### 4.2. Electron Beam Lithography (EBL)

EBL is a nanofabrication technique used in the production of microelectronic devices, ICs, and other nanoscale structures [93,94,95]. It is a high-resolution patterning method that utilizes a focused beam of electrons to write patterns on a substrate. In EBL, a beam of electrons is generated using an electron source, typically a heated filament or a field emission source. The electron beam is then accelerated and focused using electromagnetic lenses, which help to achieve a fine beam spot size in the range of a few nanometers. The substrate, often made of a material such as silicon or a semiconductor wafer, is coated with a resist material sensitive to the electron beam. The resist is typically a polymer that undergoes a chemical change when exposed to the electron beam [96].

To create patterns, the focused electron beam is scanned across the resist-coated substrate in a predetermined pattern. As the beam interacts with the resist, it causes chemical changes in the exposed areas. These changes can be either a physical alteration of the resist or a chemical reaction that modifies its properties. After the electron beam exposure, the resist is developed using a suitable solvent, selectively removing the exposed or unexposed regions of the resist, depending on the resist type (positive or negative resist). The remaining resist forms a mask that can be used for subsequent processes such as etching or deposition of materials [97]. EBL finds applications in various fields, including microelectronics, photonics, nanotechnology, and research laboratories where precise control of nanoscale features is required [98,99].

EBL offers several advantages over other lithographic techniques, such as high resolution, sub-10 nm patterning capability, and the ability to create complex patterns [100]. However, it is a relatively slow process compared to optical lithography and is often used for low-volume or specialized applications where a high resolution is crucial [101]. The cost efficiency of EBL is relatively low due to the need for expensive electron beam systems, sophisticated vacuum environments, and longer exposure times. These factors limit its applicability for large-scale manufacturing.

### 4.3. Nanoimprint Lithography (NIL)

NIL is a promising nanofabrication technique that enables the production of intricate patterns and structures at the nanoscale, with potential applications in various fields requiring precise and miniaturized features [102,103]. It is a process that involves the replication of a template or mold onto a substrate, resulting in the transfer of the pattern onto the substrate surface. The basic principle of nanoimprint lithography is similar to traditional lithographic techniques but with a different approach to pattern transfer [104]. In NIL, a template, typically made of a rigid material such as quartz or silicon, contains the desired pattern [105]. The template is then pressed into a polymer material that is applied to the substrate [106]. The polymer is typically in a liquid or viscous state at the imprinting temperature. During the imprinting process, pressure is applied to force the polymer to conform to the shape of the template. This is followed by a curing step, where the polymer is solidified or crosslinked to maintain the imprinted pattern. After the curing step, the template is separated from the polymer, leaving the desired pattern imprinted on the substrate [107].

NIL offers several advantages over other nanofabrication techniques. It can achieve high-resolution patterning, down to the sub-10 nanometer scale, and can be used to create both 2D and 3D structures. It is a relatively simple and cost-effective process compared to techniques such as EBL. Additionally, NIL is a high-throughput technique, allowing for the fabrication of large areas with high pattern fidelity. Applications of nanoimprint lithography include nanoelectronics, photonics, data storage, microfluidics, and biotechnology. It is used in the fabrication of nanoscale devices such as transistors, light-emitting diodes (LEDs), optical gratings, and microarrays [108]. NIL has also been explored for its potential in creating nanoscale patterns for surface functionalization, such as anti-reflection coatings or bioactive surfaces. For the creation of photonic crystal (PhC) structures for use in the visible range, NIL was used [109]. To evaluate the constructed system, PhCs were incorporated into waveguides. The holes in the PhCs are 80 nm in size, compared to the waveguides’ up to 50 µm in size. PhC formations and the associated waveguides may be accurately duplicated thanks to parameter optimization [109].

### 4.4. Laser Lithography (LL)

LL is a technique used in various fields, such as microelectronics, nanotechnology, and optics, for high-precision patterning and fabrication of structures on a small scale [110,111]. It involves using a laser beam to selectively expose or modify a material, typically a photosensitive material called a PR, to create intricate patterns or features. The resolution of LL is generally limited by diffraction effects. Even with advanced techniques like two-photon polymerization, the achievable resolution is typically limited to the sub-micrometer range. This may be insufficient for certain applications that require nanoscale features or extremely high-resolution patterning. The minimum possible feature size that can be manufactured in LL is proportional to the wavelength of light used. The feature sizes achievable are also dependent on the quality and availability of focusing optics and laser beam quality [112]. LL offers high resolution, accuracy, and flexibility, making it a valuable tool in the fabrication of microelectronic components, integrated circuits, photonic devices, microelectromechanical systems (MEMSs), and other nanostructures. The specific details of the process may vary depending on the application and equipment used [113].

LL typically requires precise focus control to achieve sharp and well-defined features. However, the depth of focus, which is the range over which the focus remains acceptable, can be limited. This can pose challenges when working with three-dimensional structures or uneven substrates. Some LL techniques are primarily optimized for specific types of substrates, such as silicon wafers or glass slides. The compatibility with other materials or unconventional substrates may be limited. Adhesion issues, chemical interactions, or thermal considerations can arise when working with different materials. The equipment required for LL can be expensive, especially for high-resolution and large-scale applications. The laser source, optics, PR materials, and other consumables contribute to the overall cost. Additionally, the complexity of the process and the need for specialized facilities can further increase the expenses.

### 4.5. X-Ray Lithography (XRL)

XRL is a high-resolution lithographic technique used for patterning on a microscopic scale. It utilizes X-rays, which have shorter wavelengths than visible light, to expose a photosensitive material and create intricate patterns [114]. XRL offers advantages in terms of resolution and pattern fidelity compared to optical lithographic techniques. XRL requires a high-intensity and collimated X-ray source. Synchrotron radiation, generated by accelerating electrons in a circular path, is commonly used due to its high intensity and tunable wavelength. A mask, similar to those used in UVL, is created with the desired pattern. However, in XRL, the mask typically consists of a high-atomic-number material, such as gold or tungsten, that can absorb X-rays effectively [115]. The X-ray mask is placed close to a photosensitive material, typically a polymer or a PR, coated on a substrate. X-rays pass through the transparent regions of the mask, exposing the underlying PR. The X-rays are absorbed by the mask’s opaque regions, preventing exposure. After exposure, the substrate with the PR is developed and etched, similar to other lithographic processes.

X-rays may produce smaller details and have greater resolutions because of their shorter wavelengths than visible light. Because of this, XRL is appropriate for applications needing incredibly precise patterning [116]. XRL, which operates on a bigger scale than EBL and can cover a rather broad region in a single exposure, offers a wider field of vision. Low diffraction in X-rays makes it possible to produce patterns that are clear and distinct. The high resolution possible in XRL is a result of this characteristic. In applications like high-aspect-ratio structures and MEMS devices, the ability to generate straight-walled features with almost vertical sidewalls is crucial [117].

Synchrotron radiation as an X-ray source requires specific infrastructure and facilities, which can be expensive and scarce. The necessity for materials that efficiently absorb X-rays makes it difficult and costly to create X-ray masks with high aspect ratios and resolution characteristics. For pattern integrity, it is essential to achieve exact alignment between the X-ray mask and the substrate. Any misalignment might cause a pattern to distort or to register incorrectly. The restricted depth of focus of XRL, like that of UVL, might be problematic when working with three-dimensional structures or uneven substrates.

By using RIE and XRL on an air/GaAs/AlGaAs asymmetric waveguide, two-dimensional photonic crystals (2D-PhC) have been created [118]. Utilizing X-ray diffraction effects and the resists’ nonlinear response, the lattice unit cell’s form has been altered during the development process. A resolution of 50 nm was achieved while fabricating rings, either with or without a central pillar. A well-defined photonic band structure and band anti-crossing are both present, according to optical characterization that has been carried out. The findings were reviewed, and the photonic band dispersion calculations were contrasted with the data. Photonic modes with tiny line widths and minimal propagation losses were demonstrated to be produced by structures with large dielectric fractions. This lithographic process is helpful for a thorough examination of optical characteristics’ dependency on the lattice unit cell shape because of the dependability and exact control of the constructed sample structures [118].

### 4.6. Ion Beam Lithography (IBL)

IBL is a nanoscale lithographic technique that utilizes focused ion beams (FIBs) to pattern materials with high precision. It offers the ability to create complex and three-dimensional structures at the nanometer scale. Ion beam lithography is commonly used in research and development settings for prototyping, device fabrication, and material characterization [30,119]. IBL requires an FIB source, typically based on a gallium (Ga^+^) ion beam. The beam can be generated by ion sources, such as liquid metal ion sources (LMISs) or plasma sources. The substrate, often a silicon wafer or a thin film, is prepared by cleaning and coating it with a PR material that is sensitive to ion beam exposure. A digital pattern is designed using CAD software, specifying the desired pattern to be created on the substrate [120]. The FIB is scanned over the substrate surface, following the pattern defined by the CAD design. The beam is precisely focused to a spot size of a few nanometers, allowing for high-resolution patterning. As the ion beam is scanned over the substrate, it selectively removes or modifies the PR material. The ions interact with the PR, leading to processes such as sputtering, implantation, and chemical reactions that result in the desired pattern transfer. After ion beam exposure, the substrate is subjected to a development process to remove the exposed or modified resist. Depending on the application, further etching or deposition steps may be performed to transfer the pattern into the underlying material or add functional layers [121].

Ion beams can achieve a sub-nanometer resolution, enabling the fabrication of extremely fine features and complex structures with high precision [122]. IBL allows for the fabrication of three-dimensional structures by controlling the ion beam incidence angle and beam energy. This capability is particularly useful in applications such as nanofluidics, photonic crystals, and MEMS devices. IBL is a direct-write technique, meaning that patterns can be created by directly scanning the ion beam over the substrate without the need for masks or PR exposure steps. IBL can be applied to a wide range of materials, including metals, semiconductors, polymers, and insulators, making it versatile for various research and development applications [123].

IBL is widely used in academic and research settings for nanoscale device fabrication, materials’ characterization, and fundamental research in nanotechnology. It is particularly valuable for prototyping and exploring new device concepts due to its high resolution. IBL is typically slower compared to other lithographic techniques due to the serial nature of the direct-write process [124]. It may take longer to pattern large areas or complex structures. IBL equipment can be expensive and requires specialized infrastructure, including a high-vacuum environment and sophisticated beam control systems. The interaction between the ion beam and the substrate can lead to sample damage, such as ion implantation and material displacement, which may require additional processing steps for repair or mitigation [125]. Table 1 summarizes the vital characteristics of UVL, IBL, EBL, NIL, LL, and XRL processes.

### 4.7. Scanning Probe Lithography

Scanning probe lithography (SPL) is a family of high-resolution patterning techniques used in nanofabrication, which leverages the fine control and sharp tips of scanning probe microscopy (SPM) to create precise features at the nanoscale. SPL operates by moving a sharp probe, typically a conductive or mechanical tip, over the surface of a substrate, manipulating it to create desired patterns with remarkable precision. SPL methods are capable of patterning features with dimensions ranging from several nanometers down to the atomic scale, making it a key tool in areas such as semiconductor manufacturing, molecular electronics, and biological applications [126].

The fundamental working principle of SPL relies on the interaction between the scanning probe and the substrate’s surface. The probe is generally mounted on a cantilever, like those used in Atomic Force Microscopy (AFM), and can interact with the surface in different ways to either remove material, deposit material, or modify the surface’s properties. By controlling the position of the tip with nanometer precision and using feedback mechanisms to monitor the tip-surface interaction, SPL can generate highly accurate and reproducible nanoscale features. The resolution of SPL is determined largely by the radius of the probe tip, which can be as sharp as a few nanometers or even atomic in certain cases.

There are several variations of SPL, each employing different physical or chemical mechanisms for pattern generation. Some prominent SPL techniques include the following:

(a) Dip-Pen Nanolithography (DPN): In DPN, the probe tip acts like a quill pen, depositing molecules (such as ink) onto the surface as it moves. This method relies on capillary forces between the tip and the substrate to transfer material, allowing for localized chemical patterning. DPN is especially useful for biological and chemical patterning, where precise molecule placement is critical [127,128].
(b) Nanoshaving/Nano-oxidation: This method involves physically removing material from the surface (nanoshaving) or locally oxidizing the surface (nano-oxidation) to create patterns [129]. In nano-oxidation SPL, the probe applies a voltage to the surface, which induces an electrochemical reaction, altering the material’s structure. This is commonly used on semiconductor surfaces, metals, or oxides to create fine features [130].
(c) Thermal Scanning Probe Lithography (t-SPL): In this technique, a heated probe tip melts or softens a polymer resist material on the surface, allowing for precise material removal through thermal energy [131]. The t-SPL process allows for a sub-10 nm resolution and is advantageous in applications requiring rapid, maskless patterning at nanoscale dimensions [132].
(d) Conductive Atomic Force Microscopy (C-AFM): This version of SPL uses a conductive tip to apply a localized electric field that either causes a breakdown in the material or induces deposition, allowing for electrical patterning at a nanoscale resolution [133]. C-AFM is often employed in creating nanoscale electrical circuits and devices [134].

SPL finds applications in a wide range of fields, primarily in the areas of electronics, photonics, biotechnology, and materials science. It is highly useful for developing prototypes of nanodevices, including transistors, sensors, and quantum computing elements, thanks to its ability to control features at the atomic or molecular level. In semiconductor technology, SPL can be used to create complex, multi-layered structures such as nanowires, quantum dots, and nanoscale transistors, which are integral to the miniaturization of integrated circuits. In biology and biochemistry, SPL has been employed to pattern biomolecules, cells, or proteins onto surfaces in highly controlled arrays, enabling advancements in biosensing and tissue engineering [135]. The high resolution of SPL also facilitates research in fundamental physics, where researchers use it to manipulate individual atoms or molecules to explore quantum phenomena and molecular behavior at the smallest scales [136].

One of the significant advantages of SPL is its maskless nature, which eliminates the need for expensive and complex photomasks required in traditional lithography processes. SPL also allows for direct, on-demand patterning, making it ideal for rapid prototyping and small-scale production. Its versatility in patterning different materials, from organic molecules to metals and semiconductors, makes it highly valuable for research and development. However, SPL has limitations in throughput compared to conventional lithographic techniques like photolithography or electron-beam lithography, which are better suited for large-scale industrial applications. Since SPL is inherently serial in nature, with patterns being written point by point, it is typically slower and less efficient for mass production. Moreover, the lifetime and wear of the probe tips can limit long-term operation and increase operational costs [137].

### 4.8. Soft Lithography

Soft lithography is an innovative and versatile microfabrication technique widely applied in fields such as microfluidics, biological sciences, and flexible electronics [138]. It involves the creation of finely detailed micro- and nanoscale patterns on a soft, elastomeric stamp, typically made from PDMS, which is molded against a master template containing the desired microstructures [139]. Once the stamp is formed, these patterns can be transferred onto various substrates through techniques like microcontact printing, replica molding, or microtransfer molding [140]. Unlike conventional photolithography, which relies on high-energy light to etch patterns into photosensitive materials, soft lithography leverages mechanical deformation, surface chemistry, and capillary forces to achieve patterning with high precision [141]. This makes the process highly cost-effective, accessible, and flexible, as it eliminates the need for expensive clean-room environments and complex exposure equipment [142,143].

Soft lithography also allows for patterning over large areas, on curved or flexible substrates, and with a wide variety of materials—including polymers, biological molecules, and hydrogels—expanding its applicability across numerous domains. Its capacity to integrate biological or soft materials with electronic systems further positions it as a key tool for developing next-generation technologies, such as biosensors, lab-on-a-chip devices, and flexible electronic circuits [144]. Moreover, soft lithography’s adaptability to non-planar surfaces and low-temperature processes makes it particularly suited for the fabrication of wearable and stretchable devices [145].

Soft lithography is becoming increasingly popular for producing low-cost micro- and sub-microstructures. In many applications, there is a need to fabricate structures that vary in both lateral size and height, known as hierarchical structures, on a single substrate. However, traditional microfabrication techniques, particularly photolithography, commonly used in the IC industry, struggle to achieve this. Li et al. presented a straightforward and cost-effective approach to creating hierarchical structures using capillary force lithography (CFL) [146]. The process leveraged the simple principle that the time required for a fluid to fill cavities in the mold depends on factors like the contact angle of the mold or the cavity width. Building on this concept, two methods were developed for fabricating hierarchical structures. The first involved selectively modifying the wettability of a PDMS mold through UV or oxygen plasma treatment, allowing different areas to fill at different rates. The second method created molds with patterns of varying dimensions. By employing PDMS molds with modified wettability or patterns of different sizes, hierarchical structures were successfully produced, including convex/concave microlens arrays and striped surfaces with convex/concave features [146].

### 4.9. Interference Lithography

Interference lithography is an advanced and highly precise patterning technique that harnesses the wave-like properties of light to generate periodic nanoscale structures [147]. This method is crucial in fields such as semiconductor manufacturing, photonics, and nanotechnology, where there is a constant demand for producing features that are smaller than the wavelength of visible light [148]. The fundamental principle behind interference lithography is the interference of coherent light waves. When two or more coherent beams intersect, they interfere constructively and destructively, forming a stationary wave pattern consisting of alternating bright and dark fringes. These fringes, representing regions of high and low light intensity, are projected onto a photosensitive material, such as a photoresist, which undergoes selective chemical changes when exposed to light. By fine-tuning the wavelength of light, the angle between beams, and other parameters, researchers can control the periodicity, or the spacing between fringes, as well as the dimensions of the pattern being formed. This flexibility makes interference lithography a highly efficient, scalable technique for producing intricate nanoscale patterns without the need for traditional masks or templates, making it ideal for high-volume production and advanced research applications [149].

One of the key elements of interference lithography is the use of coherent laser light, which is essential for generating a stable interference pattern. Typically, a laser beam is split into two or more paths using beam splitters and mirrors, after which the beams are recombined at controlled angles to produce the desired interference pattern. The periodicity of the interference pattern is determined by the angle between the beams and the wavelength of the light, with wider beam angles producing finer features and smaller angles resulting in larger pattern dimensions. For instance, a small angle between the beams might create patterns with larger periodic features, while increasing the angle results in much finer, nanoscale structures. This level of control makes interference lithography exceptionally versatile. It enables the fabrication of large-scale, uniform patterns over wide areas, a significant advantage over conventional photolithography, which requires complex mask fabrication and alignment processes. The ability to pattern over large areas with minimal defects also makes this method especially suitable for applications such as the creation of photonic crystals, diffraction gratings, and nanostructured surfaces that require high levels of precision and uniformity [150].

A major advantage of interference lithography is its inherent scalability and cost-effectiveness. Because it does not rely on physical masks, the process avoids the limitations and expenses associated with mask-based lithographic techniques. This maskless nature also eliminates the need for intricate alignment procedures, making the process faster and more efficient [150]. Additionally, interference lithography can be employed to pattern large substrates with minimal effort, making it highly suitable for industrial applications where high throughput is essential. The technique can also be adapted to create 3D nanostructures through multi-beam interference setups or by conducting multiple exposures at different angles, thereby enabling more complex and functional architectures. Furthermore, by manipulating various parameters, interference lithography can produce hierarchical patterns, combining different feature sizes and periodicities in a single process. This makes it ideal for fabricating advanced optical devices like metamaterials, waveguides, and plasmonic structures, where control over both surface morphology and optical properties is crucial [151].

Despite its many advantages, interference lithography is not without challenges. One of its primary limitations is its restriction to periodic patterns, which makes it unsuitable for fabricating arbitrary or highly intricate shapes. This limitation can be addressed by combining interference lithography with other lithographic techniques, such as electron-beam or focused ion beam lithography, but doing so increases the complexity and cost of the process [152]. Another significant challenge is the technique’s sensitivity to environmental factors. Vibrations, air turbulence, and even minor fluctuations in the laser’s output power can negatively impact the quality of the interference pattern, leading to defects and inconsistencies in the final structure. Additionally, while interference lithography can produce features smaller than the wavelength of light due to the nature of interference, it is still fundamentally constrained by diffraction limits. This becomes especially problematic when pushing for sub-100 nm feature sizes, as achieving these dimensions requires either shorter wavelengths, such as EUV light, or other enhancement techniques, which may introduce additional complexity and cost [153].

### 4.10. Colloidal Lithography

Colloidal lithography is a powerful nanofabrication technique that leverages the self-assembly properties of colloidal particles to create nanoscale patterns on substrates. It is an attractive alternative to traditional top-down lithographic methods such as EBL and photolithography, which are often expensive and time-consuming and require highly sophisticated equipment [154]. Colloidal lithography, in contrast, is cost-effective, relatively simple to implement, and capable of producing large-scale, ordered nanostructures. These characteristics make it ideal for applications in various fields, including optoelectronics, plasmonics, metamaterials, and even bioengineering [155,156].

The process of colloidal lithography typically begins with the synthesis of colloidal particles. These particles are usually made from materials like polystyrene, silica, or latex and are highly monodisperse, meaning they have a uniform size and shape [157]. Particle sizes can vary widely, from a few nanometers to several micrometers, depending on the desired feature size of the final nanostructures [157]. Once synthesized, these colloidal particles are deposited onto a substrate, forming a close-packed, hexagonal lattice. The method of deposition can vary depending on the desired packing density and uniformity. Common techniques include spin-coating, where the colloidal suspension is dropped onto a spinning substrate to spread it uniformly, and dip-coating, where the substrate is immersed in the colloidal suspension and then withdrawn at a controlled rate, allowing the particles to self-assemble into a monolayer [158].

Once the colloidal particles are in place, they serve as a physical mask for subsequent lithographic processes. These can include metal deposition (such as gold, silver, or aluminum), RIE, or plasma etching. For instance, in metal deposition, the metal is evaporated onto the substrate, accumulating in the gaps between the particles. After the deposition, the colloidal particles can be removed, leaving behind an array of metal nanostructures, such as nanodots, nanoholes, or nanowires, depending on the geometry of the initial colloidal mask [158]. Another approach is to use the colloidal particles themselves as an etch mask, where the uncovered regions between the particles are etched away, creating nanopillars or other features on the substrate surface.

Several variations of colloidal lithography have been developed to enhance the flexibility and versatility of the technique:

(a) Nanosphere Lithography (NSL): This is one of the most widely used forms of colloidal lithography, where a monolayer of spherical colloids (nanospheres) is assembled on a substrate, and metal deposition is performed at normal or oblique angles [159]. The subsequent removal of the colloidal spheres results in a periodic array of metallic nanostructures [160].
(b) Hole-mask Colloidal Lithography (HCL): In this method, a colloidal monolayer is used as a mask during metal deposition, but instead of removing the colloids immediately, they are embedded in a secondary mask (like a polymer layer) [161]. After the removal of the colloidal particles, the holes left behind in the polymer serve as a template for further lithographic steps, allowing for more complex patterning than traditional NSL [162].
(c) Multilayer Colloidal Lithography: By stacking multiple layers of colloidal particles or using particles of different sizes, more complex and hierarchical nanostructures can be achieved [163]. This approach opens up possibilities for fabricating 3D nanostructures, which are of particular interest in fields like photonics and plasmonics [164].
(d) Non-spherical Colloidal Lithography: Most colloidal lithographic techniques utilize spherical particles because they naturally form hexagonal close-packed arrays [165]. However, non-spherical colloids, such as ellipsoids, rods, or cubes, can be used to create more diverse and anisotropic patterns. This has potential applications in advanced optical materials and meta-surfaces, where directionally dependent properties are desired [166].

Colloidal lithography has a wide range of applications due to its ability to produce well-ordered, periodic nanostructures. In plasmonics, for example, the metallic nanostructures fabricated via colloidal lithography can act as plasmonic antennas that enhance local electromagnetic fields, making them useful in applications such as SERS, biosensing, and nano-optics [163,167]. Similarly, in photonic crystals and metamaterials, colloidal lithography can be used to create structures with a tailored refractive index, allowing for the manipulation of light in novel ways, including the creation of negative index materials or perfect lenses. In biosensing, the high surface-area-to-volume ratio of nanostructures produced via colloidal lithography enables enhanced interaction with biomolecules, leading to increased sensitivity in detecting small amounts of biological analytes. The technique also finds an application in the fabrication of superhydrophobic surfaces, where the nanostructures produced mimic the surface of lotus leaves, creating surfaces that repel water due to their hierarchical roughness.

Colloidal lithography offers several key advantages over traditional nanofabrication methods. Its most prominent benefit is scalability. Large-area nanostructured surfaces can be produced relatively quickly and inexpensively, making it suitable for industrial-scale applications. Additionally, the method is highly versatile, as the size, shape, and arrangement of the colloidal particles can be easily tuned, allowing for customization of the nanostructures produced. This makes it highly attractive for research in fields requiring precise control over nanoscale features, such as nanophotonics and materials science [168].

However, colloidal lithography also has limitations. One of the main drawbacks is the difficulty in controlling the positions of individual colloidal particles with nanometer-scale precision. While the self-assembly process can produce highly ordered arrays, it is generally limited to simple, periodic patterns. More complex and arbitrary nanoscale designs are not easily achievable with colloidal lithography alone, and integration with other lithographic techniques may be required. Another challenge is maintaining uniformity over very large areas, especially if there are defects or variations in the packing of the colloidal monolayer. This can lead to inhomogeneities in the final nanostructures, which can be problematic for certain applications requiring highly precise and defect-free surfaces [169].

### 4.11. Plasma Lithography

Plasma lithography is an advanced fabrication technique used in micro- and nanoscale patterning of materials, particularly in the semiconductor and nanotechnology industries [170]. It leverages plasma—an ionized gas containing free electrons, ions, and neutral particles—to etch or deposit material on a substrate with high precision. Plasma lithography is a crucial process for creating intricate structures on integrated circuits, microchips, and other nanodevices, enabling the continued miniaturization of electronic components according to Moore’s Law. The process of plasma lithography involves a plasma source, usually derived from gases like oxygen, chlorine, or fluorocarbons, which is exposed to the substrate to create desired patterns [171]. The plasma is created by applying an electric field to a gas under low pressure, energizing the gas molecules and generating reactive species. These reactive species can interact with the material on the substrate in various ways, depending on whether the process is focused on etching (removal of material) or deposition (adding material). In etching, the ions and radicals from the plasma chemically react with the material on the surface, removing it selectively where the plasma is directed [172].

What distinguishes plasma lithography from conventional photolithography is the use of a plasma environment to define patterns, rather than relying on light and photomasks alone. This approach allows for more flexibility and control over etching profiles, as well as improved resolution due to the interaction of high-energy plasma species with the substrate [173]. Plasma-based processes also allow for anisotropic etching, which creates more vertical sidewalls, a critical requirement for deep nanoscale patterning. There are various types of plasma lithographic techniques, with the most common being plasma etching and RIE. Plasma etching uses chemically reactive plasma to remove material, while RIE combines physical sputtering (from ion bombardment) and chemical etching, allowing more precise control over the etching depth and profile. In RIE, the substrate is exposed to both reactive ions and neutral species, with the ions bombarding the surface to physically assist in the removal of material [174].

Another advanced variant is ICP etching, which uses inductively generated plasma to enhance the density and energy of reactive species, resulting in faster and more uniform etching processes. This is particularly important when dealing with deep, narrow features that require high aspect ratios, such as those found in advanced semiconductor devices [175].

Plasma lithography has become a key tool in semiconductor manufacturing, where it is used to create features with sizes as small as a few nanometers. The technique is essential for patterning complex ICs, MEMSs, and various nanostructures used in sensors, optical devices, and even biochips [170]. Its ability to provide a high resolution and control over etching depth makes it a preferred choice for industries pushing the limits of miniaturization and performance in electronics [176]. One of the main advantages of plasma lithography is the anisotropic etching capability, which is critical for creating well-defined, vertical features that are essential in modern transistors and interconnects. Additionally, plasma processes can work with a wide variety of materials, including silicon, metals, and polymers, offering versatility across different fabrication needs. Moreover, plasma lithography offers greater control over the etching environment, such as tuning the gas composition, pressure, and power, allowing fine adjustments to achieve the desired pattern fidelity [177].

## 5. Concluding Remarks and Recommendations

GSL is a low-cost, one-step lithographic technique that uses an optical grayscale mask to generate 3D microstructures in a photoresist layer. During the lithography process, UV-light passing through the grayscale mask generates local intensity modulation, and the 3D profile on the surface of the PR stays on the substrate after the exposed PR has been removed by development. This is due to the photoactive component absorbing varied light energy as the light passes through the PR. However, GSL without a mask offers more creative freedom when designing detailed and complicated designs. Masked lithography restricts the design to the predetermined patterns on the mask. On the contrary, using maskless lithography, the substrate may be directly written or projected with grayscale patterns, giving more creative flexibility in the design and fabrication of different structures.

GS-DWL offers an unparalleled flexibility and versatility when it comes to crafting intricate patterns. This cutting-edge technology eliminates the reliance on masks or photomasks, liberating your creative potential. With dynamic control over the laser beam, GS-DWL allows for directly imprinting patterns onto the substrate, making it an ideal choice for swift prototyping and tailor-made designs. The laser systems integrated into GS-DWL boast an exceptional spatial resolution, enabling the precision fabrication of minute details and fine features. This resolution hinges on the laser spot size and the exactitude of the scanning system, ensuring the designs come to life with the utmost precision and clarity. This capability is crucial in applications that require high precision and small-scale structures, such as microelectronics, photonics, and microfluidics. GS-DWL is well-suited for rapid prototyping and iterative design processes. The ability to directly write patterns eliminates the need for fabricating masks, reducing the turnaround time and cost associated with design iterations. This facilitates quick modifications, testing, and optimization of designs.

GSL based on DMD enables the fabrication of high-resolution patterns. The DMD device consists of an array of micromirrors, each capable of independently controlling the amount of light reflected. By varying the mirror tilt angles, grayscale levels can be achieved, allowing for precise control over the exposure dose and enabling the creation of complex patterns with a high resolution. Traditional lithographic techniques typically require multiple photomasks or multiple exposures to achieve grayscale effects. GSL based on DMD eliminates the need for multiple masks and simplifies the fabrication process. It reduces the number of process steps, thereby saving time and reducing costs associated with mask fabrication and alignment.

Considering that EBL has been established as a well-proven technique for two-dimensional nanofabrication, it emerges as a strong contender for grayscale direct nanopatterning. Over the course of several decades, EBL has seen significant advancements, with most of the inherent physical challenges now addressed by state-of-the-art EBL machines, sophisticated software solutions like BEAMER by GenISys (GenISys GmbH, Munich, Germany), and innovative development methods. However, GSL extends the boundaries of this method significantly. In addition to the usual challenges related to charging on non-conductive surfaces, grayscale patterning with EBL becomes exceedingly intricate due to proximity effects, which further complicate the computation of three-dimensional patterns. Moreover, when precision at the nanometer scale is crucial, the necessary wet chemical development becomes exceptionally challenging. The resulting 3D nanostructures can even exhibit variations across the same substrate due to the slightest fluctuations in temperature and time. However, the selection of a lithographic method hinges on the precise requirements and limitations of the intended application. GSL, for instance, empowers the creation of intricate 3D structures with an unparalleled level of precision. By adjusting the exposure dose, it offers the flexibility to generate various heights or depths within a single layer. This adaptability positions GSL as an ideal choice for applications like micro-optics, microfluidics, and MEMSs, where the capability to craft highly detailed 3D structures is paramount. One of GSL’s most notable advantages lies in its ability to obviate the necessity of multiple lithography steps or intricate patterning techniques. This means that it achieves the desired 3D structures in a single exposure step, streamlining the overall process and concurrently curtailing manufacturing time and costs.

While GSL presents a remarkable array of benefits, it is essential to acknowledge its inherent limitations. Notably, GSL may not be the optimal choice for applications demanding exceptionally high aspect ratios or involving extensive large-scale production. Moreover, the GSL process itself can introduce complexities, necessitating specialized equipment or materials. In short, GSL excels in applications requiring intricate 3D structures, exceptionally high resolutions, and a flexibility of customization. Nevertheless, one must carefully assess the specific needs and constraints of each application to select the most suitable lithographic method. In alternative scenarios, conventional UVL or deep UVL may prove more fitting for the task at hand.

## Figures and Tables

**Table 1:** Comparison of vital characteristics of different lithographic methods.

	UVL	IBL	EBL	NIL	LL	XRL
Resolution	Sub 100 nm	Sub 10 nm	Sub 5 nm	Sub 10 nm	200 nm–300 nm	Sub 10 nm
Processing speed	High	Low	Low	High	Moderate	Low
Cost-effectiveness	Moderate	Low	Low	High	Low	Low

## Data Availability

Not applicable.

## Abbreviations

Integrated circuit = IC; computer-aided design = CAD; design rule checking = DRC; optical proximity correction = OPC; ion beam lithography = IBL; laser lithography= LL; ultraviolet lithography = UVL; electron beam lithography = EBL; nanoimprint lithography = NIL; X-ray lithography = XRL; diffractive optical elements = DOEs; photoresist = PR; digital micromirror device = DMD; microelectromechanical systems = MEMSs; optical proximity correction = OPC; microlens array = MLA; photonic crystal = PhC.
